# Editorial: Translational phenomics and its applications in immunotherapy

**DOI:** 10.3389/fimmu.2023.1211704

**Published:** 2023-05-22

**Authors:** Hai Fang, Liye Chen, Leroy Hood, Qiang Tian

**Affiliations:** ^1^ Shanghai Institute of Hematology, State Key Laboratory of Medical Genomics, National Research Center for Translational Medicine at Shanghai, Ruijin Hospital affiliated to Shanghai Jiao Tong University School of Medicine, Shanghai, China; ^2^ Nuffield Department of Orthopaedics, Rheumatology and Musculoskeletal Sciences, University of Oxford, Oxford, United Kingdom; ^3^ Hood-Price Lab, Institute for Systems Biology, Seattle, WA, United States

**Keywords:** phenomics, immunotherapy, systems biology, deep phenotyping, systems immunology, translational medicine

Immunotherapy has emerged as a promising treatment option for a variety of diseases, including cancer and immune-mediated disorders. Its success depends on identifying the right target, selecting the right patient population, monitoring host immune responses, and managing adverse effects. Coined by Steven Garan (1), the term “phenomics” (2) refers to all measurable micro- and macro-phenotypic manifestations beyond an individual’s genome, including cellular senescence (3), the tumor microenviroment (4), metabolome (5), and gut microbiome (6), to name but a few. Deep phenotyping of the patient immune system and diseased tissues is essential to better understand disease pathogenesis, identify key diagnostic biomarkers, and develop rational therapeutics with favorable clinical outcomes (7–9). The Frontiers in Immunology Research Topic “Translational Phenomics and its Applications in Immunotherapy” has published cutting-edge articles that use systems thinking and phenomics strategy (8, 10), along with omics and 3D printing technologies, to address significant challenges in immunotherapy. This Editorial summarizes six original articles included in this Research Topic, which can be grouped under three umbrellas, namely, “cellular senescence and cancer immunotherapy”, “rational treatments and diagnostic markers”, and “omics strategies and 3D printing technologies”.

## Cellular senescence and cancer immunotherapy

### Unlocking the power of cellular senescence-related genes: prognostic and therapeutic significance in clear cell renal cell carcinoma

This study conducted a comprehensive analysis of the significance of cellular senescence-related genes in ccRCC and their impact on patient survival and treatment response (Lu et al.). By leveraging public databases, the authors identified three molecular subtypes of ccRCC with varying clinical manifestations, mutation patterns, and tumor microenvironments. They also developed a risk model based on five cellular senescence-related genes that accurately predicted patient response to immunotherapy and chemotherapy. These findings provide a theoretical foundation for further exploration of the molecular mechanisms underlying cellular senescence-related ccRCC, with the potential of improving the risk stratification of patients. These results have important implications for the development of personalised treatment strategies for ccRCC patients.

### A cell senescence-related lncRNA signature predicts prognosis and immune response in colorectal cancer

This study focused on identifying lncRNAs linked to cell senescence and their correlations with prognosis and immune response in CRC (Xu et al.). By analysing data from TCGA and Human Aging Genomic Resources, the authors identified a signature of six cell senescence-related lncRNAs that demonstrated high prognostic value. The risk model was found to be also associated with tumor microenvironments, immune checkpoints, and drug sensitivity, with lower IC50 values observed for AICAR, cisplatin, nilotinib, and bexarotene in the high-risk group. These findings suggest that the identified cell senescence-related lncRNAs may serve as valuable biomarkers for predicting prognosis and response to immune and chemotherapy in CRC patients.

## Rational treatments and diagnostic markers

### Subgroup analysis predicts recurrence risk and personalises treatment for stage II CRC

This study developed a novel method called “TFunctionalProg” to predict the risk of recurrence and personalised adjuvant drugs for stage II CRC patients (Wang et al.). By analysing data from 222 microsatellite-stable stage II CRC patients, the authors identified two subgroups with high risk of recurrence, each exhibiting distinct phenotypes and mechanisms. Based on these findings, they proposed personalised rational adjuvant drug combinations, including immunotherapy, chemotherapy, and repurposed central nervous system drugs. This approach provides a different utility compared to circulating tumor DNA-based prognostic biomarkers, with the potential of improving the survival rate of stage II CRC patients.

### Unraveling the intricate relationship between altered gut microbiota and inflammation markers in patients with Crohn’s disease

This study investigated the correlation between altered gut microbiota and elevated inflammation markers in CD patients, using paired subjects to minimise confounding factors (Hu et al.). By analysing fecal microbiota, the authors found that CD patients had reduced levels of short-chain fatty acid producing bacteria but elevated levels of opportunistic pathogen Escherichia-Shigella. These differential genera had the potential to distinguish between CD patients and healthy controls, and showed correlations with changes in inflammation-related blood biochemical markers. The results suggest that the differential genera could be used as diagnostic markers for CD, and as potential targets for intervention, thereby paving the way for a more precise and effective treatment of the disease.

## Omics strategies and 3D printing technologies

### Prognostic signature for survival, immune microenvironment, and immunotherapy response prediction using single-cell sequencing data in patients with gastric cancers

In this study, the authors developed an immune-related genetic signature using scRNA-seq data combined with bulk RNA-seq data to predict prognosis, immune status, and response to immunotherapy in GC patients (Hu et al.). Their analysis identified nine cell types and established a signature that accurately predicted overall survival and improved clinical utility. The low-risk group had high tumor mutation burden, increased immune activation, and high microsatellite instability, indicating the suitability for immunotherapy. On the other hand, the high-risk group was associated with microsatellite stability and immunosuppression, suggesting the suitability for targeted therapy. The accuracy of the signature in predicting immunotherapeutic response was validated in an external dataset. These findings provide insight into the immune status of GC patients, prognosis assessment, and the development of efficient immunotherapeutic treatments, as well as personalised treatment strategies in the disease.

### 3D printed Ti-5Cu alloy induces M2 macrophage polarisation and accelerates osteogenic differentiation of MC3T3-E1 cells

This study investigated the effects of the Ti-5Cu alloy, prepared through 3D printing technologies, on macrophage polarisation and immune-related bone formation (Zhao et al.). The authors found that the alloy had a smoother surface compared to Ti, with no cytotoxicity observed to either RAW264.7 cells or Cu2+ at a concentration of 0.133 mg/L. The results also showed that the alloy was able to modulate macrophage polarisation towards the M2 phenotype, promoting the proliferation and osteogenic differentiation of MC3T3-E1 cells. The gene “Oncostatin M” expressed by RAW264.7 co-cultured with the Ti-5Cu alloy was found to accelerate the osteogenic differentiation of MC3T3-E1 cells through acting on its receptors. These findings highlight the potential of the Ti-5Cu alloy for accelerating osteogenic differentiation and immune-related bone formation.

## Author contributions

All authors listed have made a substantial, direct, and intellectual contribution to the work and approved it for publication.
